# Tau phosphorylation and PAD exposure in regulation of axonal growth

**DOI:** 10.3389/fcell.2022.1023418

**Published:** 2023-01-18

**Authors:** S. L. Morris, S. T. Brady

**Affiliations:** Department of Anatomy and Cell Biology, University of Illinois at Chicago, Chicago, IL, United States

**Keywords:** tau, protein phosphatase 1, fast axonal transport, kinesin, GSK3β

## Abstract

**Introduction:** Tau is a microtubule associated phosphoprotein found principally in neurons. Prevailing dogma continues to define microtubule stabilization as the major function of tau *in vivo*, despite several lines of evidence suggesting this is not the case. Most importantly, tau null mice have deficits in axonal outgrowth and neuronal migration while still possessing an extensive microtubule network. Instead, mounting evidence suggests that tau may have a major function in the regulation of fast axonal transport (FAT) through activation of neuronal signaling pathways. Previous studies identified a phosphatase activating domain (PAD) at the tau N-terminal that is normally sequestered, but is constitutively exposed in tauopathies. When exposed, the PAD activates a signaling cascade involving PP1 and GSK3β which affects cellular functions including release of cargo from kinesin. Furthermore, we discovered that PAD exposure can be regulated by a single phosphorylation at T205. Exposure of the PAD is an early event in multiple tauopathies and a major contributing factor to neurodegeneration associated with tau hyperphosphorylation. However, effects of tau PAD exposure on anterograde FAT raised the interesting possibility that this pathway may be a mechanism for physiological regulation of cargo delivery through site-specific phosphorylation of tau and transient activation of PP1 and GSK3β. Significantly, there is already evidence of local control of PP1 and GSK3β at sites which require cargo delivery.

**Methods:** To investigate this hypothesis, first we evaluated cellular localization of tau PAD exposure, pT205 tau phosphorylation, and active GSK3β in primary hippocampal neurons during development. Second, we analyzed the axonal outgrowth of tau knockout neurons following transfection with full length hTau40-WT, hTau40-ΔPAD, or hTau40-T205A.

**Results and Discussion:** The results presented here suggest that transient activation of a PP1-GSK3β signaling pathway through locally regulated PAD exposure is a mechanism for cargo delivery, and thereby important for neurite outgrowth of developing neurons.

## 1 Introduction

Neurons are polarized cells with an axon that can extend several thousand times the diameter of the cell body. Moreover the axon is not a homogeneous expanse but may include thousands of discrete subdomains, such as Nodes of Ranvier and presynaptic terminals, which have complex, specialized protein compositions. Neuron polarity and axonal subdomains are established during development of the nervous system during which neural precursors differentiate and undergo neurite outgrowth with the axon and dendrites extending outwards from the cell body. Membrane expansion is preferentially at the axon tip, which becomes progressively further from the cell body, requiring active delivery of membrane, proteins, and organelles by fast axonal transport (FAT) (Pfenninger and Friedman, 1993; Craig et al., 1995; Futerman and Banker, 1996). It has long been known that blocking axonal transport of vesicles inhibits axon growth and induces growth cone collapse (Martenson et al., 1993). Furthermore kinesin mutants, such as KIF5A^−/−^ neurons, have reduced outgrowth (Karle et al., 2012). Despite the importance of FAT and selective delivery of cargos to specific locations, the mechanisms for physiological regulation of cargo transport and delivery have remained elusive.

Tau is a microtubule associated proteins found principally in neurons. It was originally isolated with microtubules from brain and was described as essential for microtubule assembly *in vitro* (Weingarten et al., 1975). The prevailing dogma continues to define the major function of tau *in vivo* as microtubule stabilization even though several lines of evidence suggest this is not the case. Most importantly, tau null mice have deficits in neurite outgrowth and neuronal migration both *in vitro* and *in vivo* while still possessing an extensive microtubule network (Harada et al., 1994; Dawson et al., 2001; Sennvik et al., 2007; Sapir et al., 2012). Furthermore, tau expression is correlated with neuronal maturation (Fiock et al., 2020) and tau protein is enriched on dynamic microtubules at the end of growing neurites (Black et al., 1996). *In vitro*, wild-type (WT) hippocampal neurons follow a stereotypical timeline of outgrowth (Bartlett and Banker, 1984; Dotti et al., 1988; Fletcher and Banker, 1989). After a brief multipolar stage whereby several short processes grow equally, one process begins to grow rapidly to become the axon while other processes remain stalled. 2–3 days later the short processes restart growth at a slower rate elongating and branching to become dendrites (Dotti et al., 1988). Tau knockdown in primary cerebellar neurons prevented neurite elongation with cells remaining in the early multipolar stage until tau expression was reinstated (Caceres and Kosik, 1990; Caceres et al., 1991). Tau knockout hippocampal neurons also had reduced axonal and dendritic elongation (Dawson et al., 2001).

During studies evaluating biological activities of normal (Morfini et al., 2007) and pathological tau (LaPointe et al., 2009; Kanaan et al., 2011), a phosphatase activating domain (PAD) at the N-terminal of tau was identified. When exposed, the PAD activates a signaling cascade involving protein phosphatase 1 (PP1) and glycogen synthase three beta (GSK3β) (LaPointe et al., 2009; Kanaan et al., 2011). PP1 activates GSK3β by dephosphorylating pSer9 on the inactive form of the kinase. Activation of GSK3β leads to phosphorylation of kinesin light chain (KLC) and trigger the release of vesicle cargoes (Morfini et al., 2002; Kanaan et al., 2011). Exposure of the PAD has been shown to be an early event in multiple tauopathies and is a major contributing factor to neurodegeneration (Kanaan et al., 2011; Kanaan et al., 2012; Cox et al., 2016; Kanaan et al., 2016). However, the presence of a sequestered PAD in normal tau raised the possibility that this domain may have a normal physiological function. The exclusive effect of tau PAD on anterograde FAT raised the interesting possibility for physiological regulation of cargo delivery through transient activation of PP1 and GSK3β. Significantly, there is already evidence that local control of PP1 and GSK3β occurs at sites which require cargo delivery. For example, during axonal outgrowth active GSK3β is enriched at growth cones (Morfini et al., 2002; Morfini et al., 2004). Additionally, it is well known that neurofilaments are highly phosphorylated throughout axons except at nodes of Ranvier (Hsieh et al., 1994; Martini, 2001). This has been attributed to a higher activity of phosphatases in this area, but again the physiological function of this local activation and the mechanism for regulating it are unknown (de Waegh et al., 1992; Hsieh et al., 1994). We hypothesize that activation of a PP1-GSK3β signaling pathway by regulated site-specific PAD exposure is a mechanism for cargo delivery, and is thereby important for neurite outgrowth of developing neurons.

## 2 Methods

### 2.1 Animals

All experiments were performed under protocols approved by the Institutional Animal Care and Use Committee at the University of Illinois at Chicago (UIC). All animals were housed at the Biological Resource Laboratory at UIC under a 12-h light/dark cycle and provided with food and water *ad libitum*. Wild-type (WT) C57BL/6 mice (The Jackson Laboratory, #000664), Mapt^tm1Hnd^ (Tau knockout (KO)) (The Jackson Laboratory, #007251 (Dawson et al., 2001)), and Sprague Dawley rats (Charles River) were used for the experiments in this study. WT C57BL/6 or homozygous Tau KO male and female mice were paired in-house for timed pregnancies. Female timed-pregnant Sprague-Dawley rats were purchased from Charles River Laboratories.

### 2.2 Primary hippocampal neuronal culture

Embryos were collected from WT or tau knockout C57BL/6 mice at embryonic day 16.5 (E16.5), or Sprague Dawley rats at embryonic day 18.5 (E18.5). Hippocampi were dissected from both hemispheres of the brain and collected in ice-cold 1X Hanks Balanced Salt Solution (HBSS; Gibco #14185052). The tissue was dissociated in trypsin (Gibco #15140148) for 15 min in a 37°C water bath before carefully washing 3 times in 1x HBSS. Trypsin was deactivated by adding Dulbecco’s Modified Eagle Medium (DMEM; Gibco #11995065) supplemented with 10% Fetal bovine serum (FBS; Gibco #16000044) and 1X penicillin/streptomycin (Pen/Strep; Gibco #15140122) (DMEM++). The tissue was triturated into a single cell suspension with a glass Pasteur pipette followed by a flame-polished Pasteur pipette with 50% reduced diameter. The number of cells was counted with a hemocytometer and the concentrated cell solution was diluted to 1–2x10^4^ cells/well, as required, in DMEM++. Cells were plated onto 6-well μ-Slide VI 0.4 (Ibidi, #80606) chamber slides coated with 0.5 mg/ml poly-l-lysine (PLL; Sigma #P1399) in 0.1 M borate buffer and 10 μg/ml laminin (Invitrogen #23017015). After plating cells were incubated in a sterile humidified incubator at 37°C with 5% CO_2_ for 4 h at which time they underwent a 50% media change to Neurobasal™ Medium (Gibco #21103049) supplemented with 1X B27™ Plus (Gibco #A3582801), 1X pen/strep and 0.5 mM GlutaMAX™ (Gibco #35050061) (NB+++). Cells were maintained in a sterile humidified incubator at 37°C with 5% CO_2_ for the duration of the experiment with a 50% media change of fresh NB+++ every 3–4 days.

### 2.3 Transfection of primary hippocampal neurons

**FIGURE 1:**
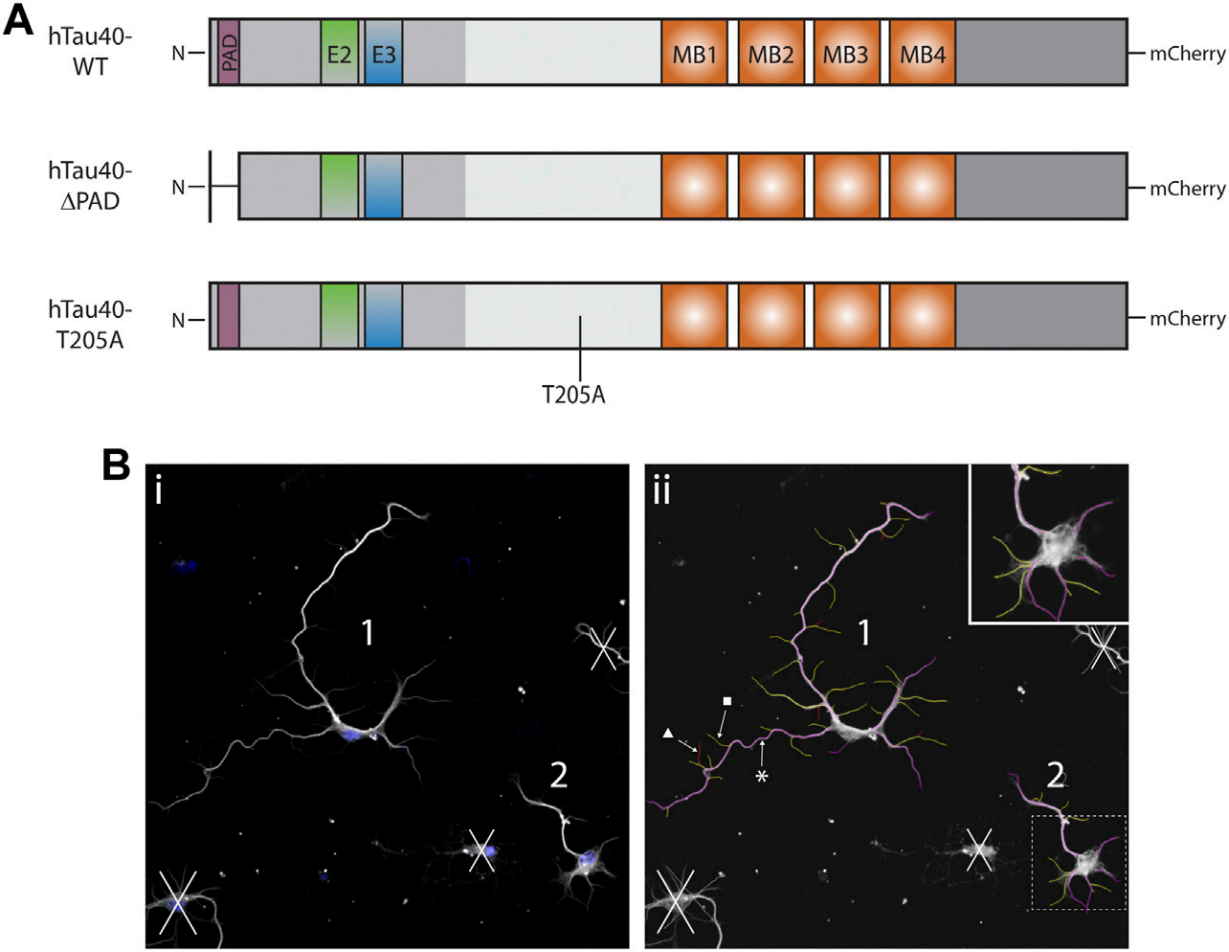
Schematics of DNA constructs used for transfection and representative outgrowth images. **(A)** Schematics of full length hTau40 DNA used for transfection of tau KO hippocampal. Neurons were transfected 16–18 h after plating and maintained in culture for a further 72 h. **(B)** 1) Representative image before neurite measurement. Blue: DAPI, White: β3-tubulin). All living neurons, judged by nucleus and tubulin integrity, fully contained in the image were identified and numbered prior to neurite measurement. 2) Representative image of tubulin immunocytochemistry after neurite measurement with the NeuronJ plugin. All neurites and branches are measured before being characterized as primary (magenta, *), secondary (yellow, ■), or tertiary (red, ▲).

For axonal outgrowth experiments, mouse primary hippocampal neurons were transfected with NeuroMag transfection reagent (OZBiosciences, #NM50500) according to manufacturer’s instructions. Neurons from tau KO mice were transfected 16–18 h after plating with hTau40-WT, hTau40-ΔPAD, or hTau40-T205A. All tau constructs included a C-terminal mCherry tag (Figure 1A). Transfection complexes were assembled by mixing 1 μL NeuroMag reagent with 0.5 μg DNA in 20 μL unsupplemented neurobasal medium. After 15 min incubation at room temperature (RT), culture media was removed from each well and gently mixed with transfection complexes before being added back onto cells. Cell culture plates were placed onto a magnetic plate (OZBiosciences, #MF10000) for 15 min at 37°C. Post-transfection, cells were maintained in an incubator until fixation for immunocytochemistry.

### 2.4 Immunocytochemistry

Primary hippocampal neurons cultured in 6-well μ-Slide VI 0.4 (Ibidi, #80606) chamber slides were fixed for immunocytochemistry. Following removal of culture medium, each well was carefully rinsed with 1X cytoskeleton buffer (10 mM MES, 138 mM KCl, 3 mM MgCl_2_, 4 mM EGTA, pH 6.1) before neurons were fixed in microtubule extraction buffer (80 mM PIPES, 2 mM MgCl_2_, 1 mM EGTA, 0.3% Triton-X) with 0.25% glutaraldehyde for 90 s followed by 4% paraformaldehyde in 1X tris-buffered saline (TBS) for 10 min. The cells were washed twice in 1X TBS for 10 min each. Fresh TBS was added and the neurons were stored at 4°C. Samples were incubated with 50 mM NH_4_Cl for 10 min at RT to quench autofluorescence then washed once with 1X TBS for 10 min before being permeabilized in 0.1% Triton-X in 1X TBS for 10 min. Neurons were blocked with 5% milk or 1% BSA in 1X TBS for 1 h at RT then rinsed twice with 1X TBS prior to incubation with primary antibodies (Supplementary Table S1) diluted in 1X TBS overnight at 4°C. The following day, neurons were washed in 1X TBS for 3 × 5 min then incubated with secondary antibodies for 1 h at RT. If required, samples were rinsed with 1X TBS then incubated with Phalloidin-AlexaFluor 647 for 30 min at RT. The samples were washed with 1X TBS for 10 min followed by a 10 min incubation with 2 μg/ml Hoechst 33342 (Thermo Scientific, #62249). The samples were again washed with 1X TBS for 10 min before ProLong Gold antifade mounting medium (Thermo Fisher Scientific, #P36930) was added to each well. The samples were stored in a parafilm-sealed 1000 mm dish wrapped in aluminum foil at 4°C until imaging.

### 2.5 Confocal imaging

The extent of colocalization for TNT1 and pT205 in growth cones and axons was evaluated by Intensity Correlation Analysis (ICA) using the JACoP plugin for ImageJ (Bolte and Cordelières, 2006). This method is a variation of Pearson’s colocalization correlation with less bias towards high staining intensities. Image stacks were separated into individual channels for analysis of green and red channels. Within the JACoP plugin, Costes’ automatic threshold was used on each image followed by Li’s ICA. In comparisons of (R_i_–R_avg_) (G_i_–G_avg_), positive values indicate colocalization while negative values represent lack of co-localization.

Following immunocytochemistry, neurons in Ibidi chambers were imaged by confocal microscopy on a Zeiss LSM 710 or Zeiss LSM 880 with Airyscan. Images were taken using a 25x, 40x or ×63 objective with oil immersion and are presented as a max projection. Images for axonal outgrowth measurements were obtained with a ×25 objective with oil immersion at a single z-plane.

### 2.6 Neurite outgrowth analysis of WT and tau KO neurons

Neurite outgrowth of WT or tau KO neurons cultured in Ibidi chambers for 24, 48, 72 or 96 h was analyzed using the ImageScience NeuronJ plugin for ImageJ. All living cells, judged by nuclear stain and tubulin integrity, contained completely within each image were numbered in ImageJ. Merged images were then split into individual channels and the tubulin channel was opened with the NeuronJ plugin. All neurites and branches were measured for one neuron before being classified as a primary, secondary, or tertiary neurite (Figure 1B). The resulting measurements were collated for analysis and the process was repeated for each neuron across all images per experiment. An average of 50 neurons were measured, across two wells, for each growth substrate and timepoint, per experiment. The following variables were analyzed for each neuron: total neurites length, longest primary neurite length, other primary neurites total length, number of primary neurites, total number of branches (secondary and tertiary neurites), total number of branches from longest primary neurite, and total length of all branches. Each variable was analyzed by one-way ANOVA with Šídák’s multiple comparisons test for individual comparisons. Significance was set at *p* < 0.05. All statistical comparisons and graphs were generated in GraphPad Prism 9.0.0.

## 3 Results

### 3.1 Exposure of tau PAD and pT205 tau are co-localized at sites of active cargo delivery similar to active dephospho-Ser9-GSK3β

In the squid axoplasm, tau PAD is both necessary and sufficient for specific inhibition of anterograde FAT through activation of a PP1/GSK3β signaling cascade (Kanaan et al., 2011). Furthermore, pseudophosphorylation of tau at T205 also inhibits anterograde FAT dependent on exposure of PAD and activation of GSK3β (Morris et al., 2021). Previous studies have shown that GSK3β can directly phosphorylate KLC and facilitate release of kinesin from a membrane bound organelle (MBO) (Morfini et al., 2002). Exclusive inhibition of anterograde FAT by this pathway led to the hypothesis that exposure of PAD at specific axonal subdomains is a mechanism for regulating MBO delivery by conventional kinesin.

**FIGURE 2:**
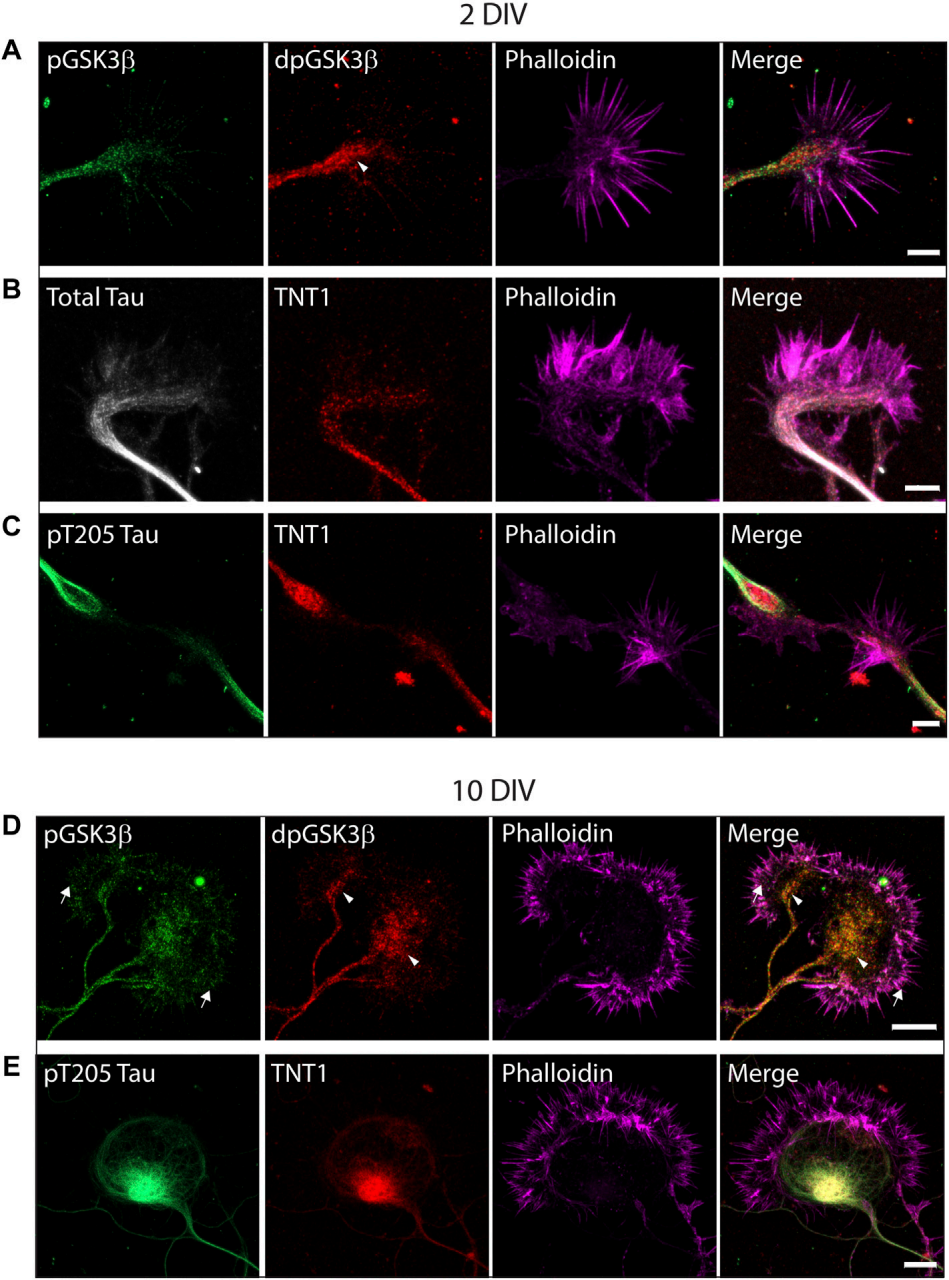
Active dp-Ser9-GSK3β, PAD exposure, and pT205 tau are localized in growth cones. WT hippocampal neurons cultured for two DIV **(A–C)** or 10 DIV **(D–E)** were immunostained for inactive phospho-Ser9-GS3β (green) and active dephospho-Ser9-GSK3β (red) **(A, D)** or TNT1 (red) **(B, C, E)**, total tau (white) **(B)** or pT205 tau (green) **(C, E)**. All neurons were also stained with phalloidin to visualize actin (magenta). **(A,D)** Active dpGSK3β is enriched in the central region of the growth cone (arrowheads) whereas inactive pGSK3β is evenly distributed along the growing neurite and throughout the growth cone (arrows). **(C, E)** At both early and late developmental timepoints, TNT1 and pT205 tau immunoreactivity are visible at the end of growing neurites and the central region of growth cones. Scale bars: 5 μm (2 DIV); 10 μm (10 DIV).

Previously it was shown that while GSK3 protein is localized in growth cones, inactive phospho-Ser9-GSK3β only comprises a fraction of total protein suggesting the presence of active dephospho-ser9-GSK3β (Morfini et al., 2002). To confirm this, primary hippocampal neurons from ∼E16.5 mice embryos were cultured for 2, 7 or 10 days before being fixed and double-stained for dephospho-ser9-GSK3β (dp-GSK3β) and phospho-Ser9-GSK3β (p-GSK3β). As shown in Figure 2, active dp-GSK3β is preferentially localized to the end of growing neurites and within the central portion of growth cones both in early outgrowth (2 days *in vitro* (DIV); Figure 2A) and later development (10 DIV; Figure 2D), which is consistent with previous reports (Morfini et al., 2002). To evaluate the exposure of tau PAD and tau phosphorylation in growth cones, primary hippocampal neurons were also stained with combinations of TNT1, pT205 tau, and total tau. Similar to active dp-GSK3β, PAD exposure (TNT1 immunoreactivity) and pT205 immunoreactivity are co-localized at the end of neurites and within growth cones at early (Figures 2B,C; Supplementary Figure S1) and later (Figure 2E; Supplementary Figure S1) developmental timepoints.

**FIGURE 3:**
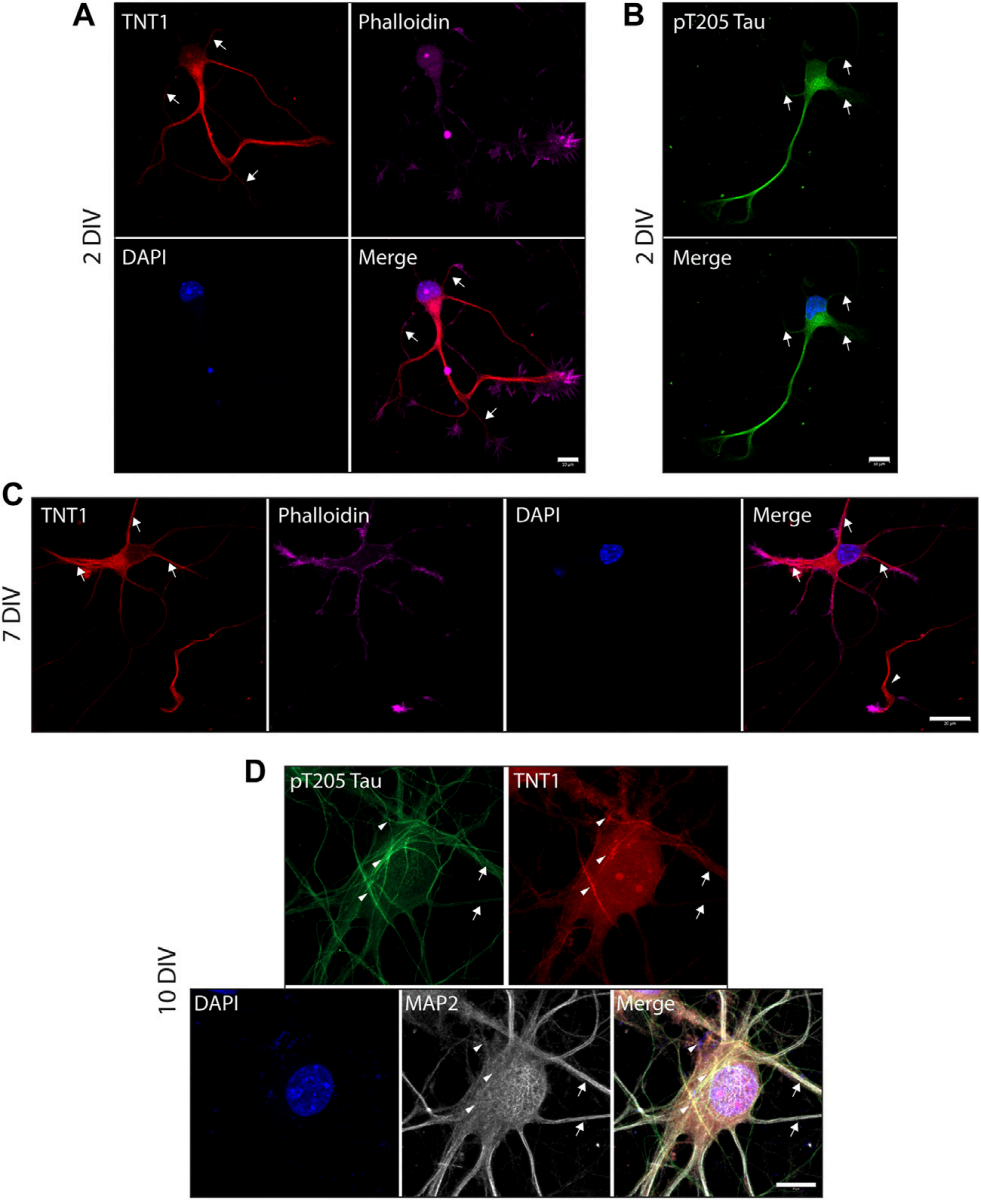
PAD exposure and pT205 tau localization changes and becomes progressively more restricted through neuronal development. WT hippocampal neurons cultured for 2 **(A, B)**, 7 **(C)**, or 10 DIV **(D)** were stained for TNT1 (red) and/or pT205 tau (green), actin (phalloidin; magenta), and MAP2 (white). At 2DIV TNT1 immunoreactivity is visible within the cell body and along the longest neurite. There is less PAD exposure in minor processes and branches (**A**, arrows). Similarly, pT205 tau immunoreactivity is equally distributed along the longest neurite and within the cell body but is not enriched in minor processes (**B**, arrows). At 7DIV, TNT1 immunoreactivity is observed in the cell body and at the end of the longest neurite (**C**, arrowhead) as well as in some minor processes (**C**, arrows). By 10DIV, PAD exposure and pT205 tau are localized to potential synaptic sites. pT205 tau and PAD exposure are localized are restricted subdomains along the axon at axonal-axonal or axonal-cell body crossing points (**D**, arrowheads). Neither pT205 nor TNT1 immunoreactivity is enriched in MAP2-positive dendrites (**D**, arrows). Scale bar**s**: 10 μm **(A, B, D) or** 20 μm **(C)**.

**FIGURE 4:**
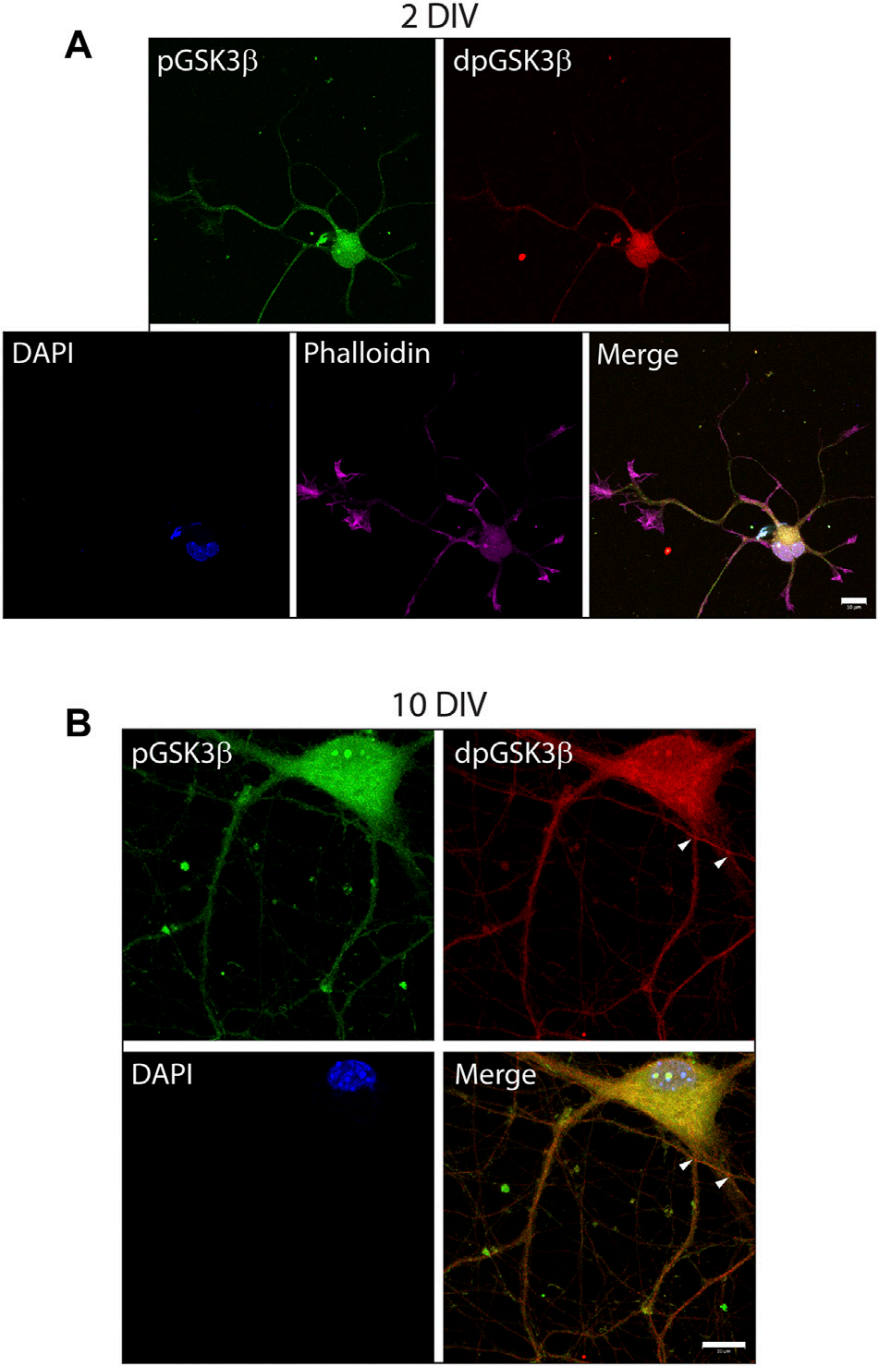
Active phospho-Ser9-GSK3β is localized at specific locations in developing neurons. WT hippocampal neurons cultured for 2 **(A)** or 10 DIV **(B)** were stained for inactive phosphor-Ser9-GS3β (green), active dephospho-Ser9-GSK3β (red), and actin (phalloidin). In early development punctate staining of inactive pGS3β is visible along the length of the growing neurites whereas active dp-GSK3β has more limited localization **(A)**. In later development, active dpGSK3β is localized at specific subdomains of the axon at points of axonal crossing (**B**, arrowheads), as well as in the cell body. Scale bars: 10 μm.

To investigate the exposure of tau PAD, T205 tau phosphorylation and the distribution of dp-GSK3β throughout the neuron at different developmental stages, whole cells were also visualized. At an early developmental timepoint (2 DIV) both TNT1 (Figure 3A) and pT205 (Figure 3B) immunoreactivity are widespread throughout the cell body and along the longest neurite, the presumptive axon. Both inactive phospho- and active dephospho-GSK3β have a similar distribution (Figure 4A). The locations of PAD exposure become progressively more restricted and by 7 DIV TNT1 immunoreactivity is most apparent at the end of growing axons (Figure 3C). This corresponds to total tau expression which is enriched in the axon, particularly in the distal axon, compared to other neuronal domains (Black et al., 1996, data not shown**)**. Interestingly, PAD exposure can also be seen in the dendrites at 7 DIV (Figure 3C, arrows). By contrast, in earlier development minor processes are devoid of TNT1 immunoreactivity (Figure 3A, arrows). In more mature neurons PAD exposure and pT205 tau are co-localized at specific sites along the axon where the axon crosses another or a cell body (Figure 3D; Supplementary Figure S1). These are potential synaptic sites which would require delivery of MBOs. Interestingly, active dp-GSK3β also appears to be more prominent at select axonal crossing points (Figure 4B).

### 3.2 Tau knockout primary hippocampal neurons have impaired axonal and dendritic outgrowth

**FIGURE 5:**
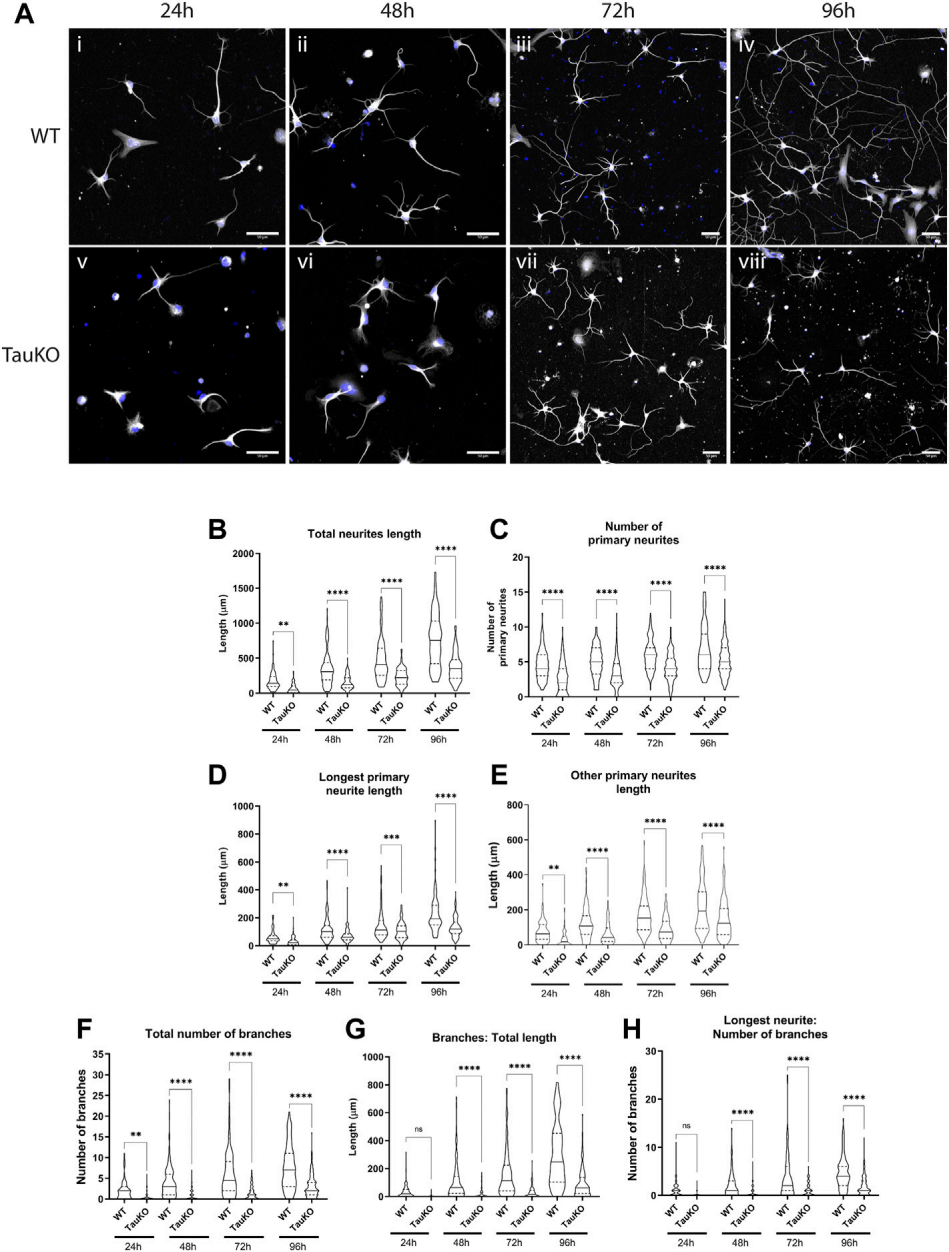
Tau KO hippocampal neurons have impaired neurite outgrowth and branching compared to WT neurons. Primary hippocampal neurons from WT and tau KO mice were cultured on poly-l-lysine + laminin for 24, 48, 72, or 96 h followed by staining for β3-tubulin. **(A)** WT hippocampal neurons (i–v) progress through the known stages of neurite outgrowth (Fletcher and Banker, 1989). By contrast, Tau KO hippocampal neurons (v-viii) have less neurite outgrowth and complexity than WT neurons at all timepoints. Blue: DAPI, White: β3-tubulin. Scale bars: 50 μm **(B)** Primary hippocampal neurons from tau KO mice have significantly shorter total neurite outgrowth length compared to WT neurons starting from 24 h of culture and continuing to 96 h of culture. **(C)** The longest primary neurite (axon) was significantly shorter for tau KO hippocampal neurons compared to WT. **(D)** The length of other primary neurites (dendrites) was significantly longer for WT hippocampal neurons compared to tau KO neurons at all timepoints. **(E)** The number of primary neurites remained consistent from 24h–96 h of culture for both WT and tau KO hippocampal neurons and was significantly for WT neurons at all timepoints. **(F)** WT hippocampal neurons have an overall higher number of axonal and dendritic branches than tau KO neurons from 24 to 96 h in culture. **(G)** Primary hippocampal neurons from tau KO mice have significantly shorter secondary and tertiary neurite lengths than WT neurons at the equivalent timepoint in culture. **(H)** WT hippocampal neurons have a significantly higher number of axonal branches than tau KO neurons. N = 96–119 individual neurons analyzed (see Supplementary Tables 2 & 3). ****p < 0.0001, ***p < 0.001, **p < 0.01, *p < 0.05.

Healthy neurite outgrowth is dependent on efficient transport of proteins and organelles to growth cones (Pfenninger and Friedman, 1993; Craig et al., 1995; Futerman and Banker, 1996). Previously it was reported that primary hippocampal neurons from tau KO mice have impaired outgrowth in culture (Dawson et al., 2001) but this finding has not been consistent between different animal models (Harada et al., 1994; Takei et al., 2000). To confirm and extend these previous studies, tau knockout neurons from ∼E16.5 mice were cultured on a PLL (Supplementary Figure S2) or PLL + Laminin (Figure 5) growth substrate. Outgrowth and neuronal morphology were analyzed by tubulin immunoreactivity after 24, 48, 72 or 96 h in culture. After 24 h, the majority of WT neurons had substantial outgrowth including one primary neurite that was visibly longer than the others and some neurite branching (Figure 5A; Supplementary Figure S2A). By contrast tau KO neurons had minimal sprouting and only diffuse tubulin staining (Figure 5A; Supplementary Figure S2A). Measurement of total neurite length confirmed that tau KO neurons had significantly less growth at all timepoints analyzed when grown on either PLL alone (Supplementary Figure S2B) or a PLL + Laminin combined substrate (Figure 5B). Similarly, the longest neurite length (axon) of tau KO neurons was significantly less than WT neurons for each timepoint and growth substrate (Figure 5D; Supplementary Figure S2D).

In addition to longer axonal outgrowth, WT neurons also had significantly more outgrowth of “other primary neurites” or dendrites at all timepoints (Figure 5E; Supplementary Figure S2E). For both WT and tau KO neurons, the number of primary neurites increased only slightly over time course of 24–96 h (Figure 5C; Supplementary Figure S2C) and were not significantly different between the growth substrates. However in all cases, the number of primary neurites was greater for WT neurons than tau KO neurons (Figure 5C; Supplementary Figure S2C). Finally, the number and length of branches were measured. After 24 h of outgrowth, some WT neurons already had up to 10 branches in total, primarily on the longest neurite, the presumptive axon (Figures 5F,H; Supplementary Figures S2F, H). The number of neurite branches continued to be significantly higher for WT neurons at every timepoint on both PLL (Supplementary Figure S2F) and PLL + Laminin growth substrates (Figure 5F). The overall length of neurite branches was also greater for WT neurons compared to tau KO neurons at every timepoint both due to the higher number of branches and longer length of each branch (Figure 5G; Supplementary Figure S2G).

In summary, in primary culture tau KO hippocampal neurons had stunted development in every measurement analyzed compared to WT hippocampal neurons. Visually, tau KO neurons were approximately 2 days behind their WT neuron counterparts (Figure 5A; Supplementary Figure S2A), confirming previous studies which showed tau is important for neurite outgrowth.

### 3.3 Impaired neurite outgrowth of tau knockout primary neurons cannot be recovered by hTau40-ΔPAD or–T205A

**FIGURE 6:**
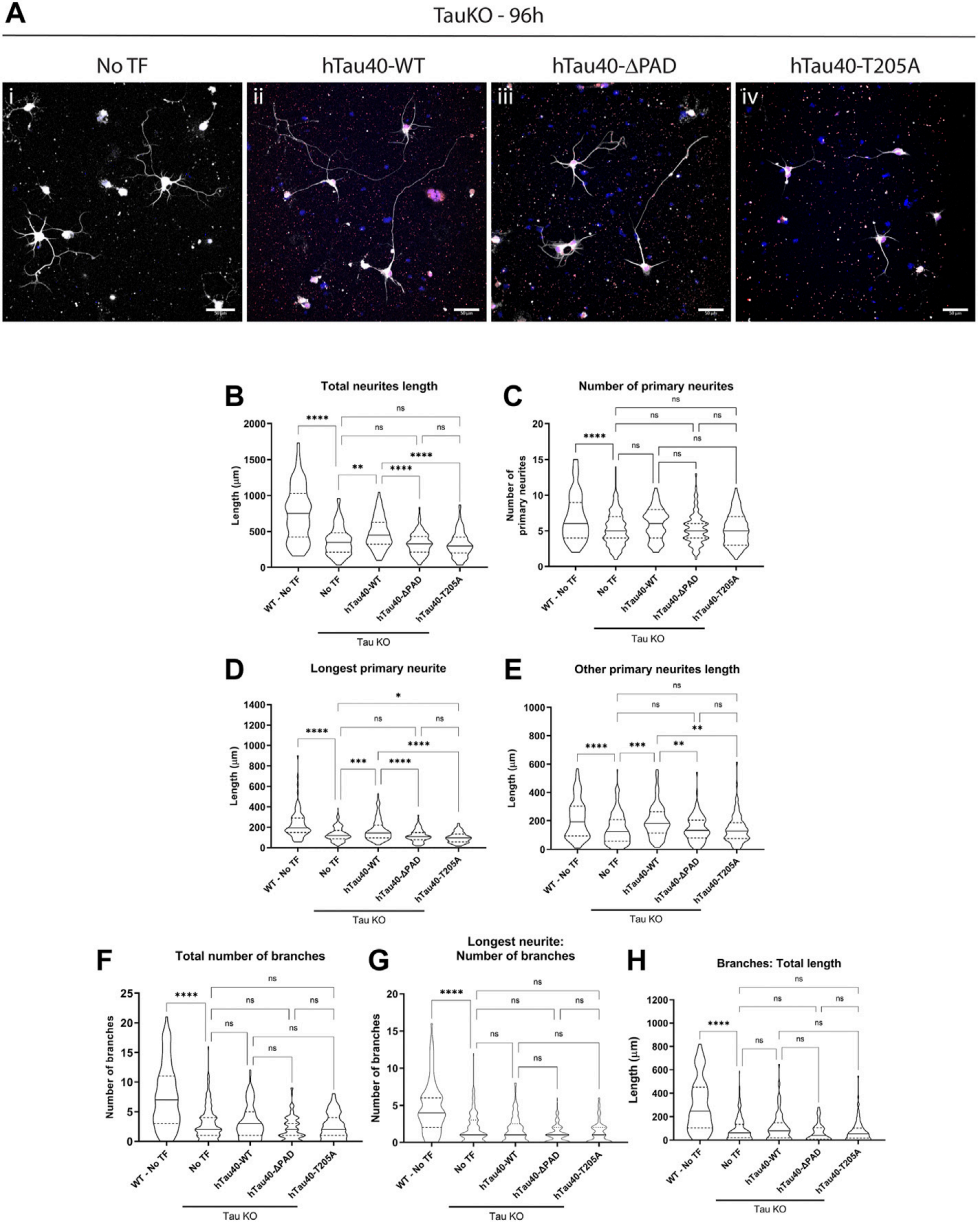
Transfection with hTau40-WT, but not with hTau40-ΔPAD and hTau40-T205A, improves neurite outgrowth of tau KO hippocampal neurons. Primary hippocampal neurons from tau KO mice cultured on poly-l-lysine + laminin were transfected 16–18 h after plating and cultured for a further 72 h before fixation and staining for β3-tubulin. **(A)** Tau KO hippocampal neurons have limited neurite outgrowth after 96 h 1) but outgrowth is improved after transfection with hTau40-WT 2). By contrast, transfection of Tau KO hippocampal neurons with hTau40-ΔPAD 3) or hTau40-T205A (iv) does not improve neurite outgrowth compared to untransfected tau KO hippocampal neurons. Blue: DAPI, White: β3-tubulin. Scale bars: 50 μm. **(B)** The total length of all neurites is increased by transfection of hTau40-WT (*p* = 0.0014) but not hTau40-ΔPAD (*p* = 0.963) or hTau40-T205A (*p* = 0.81). **(C)** The number of primary neurites for tau KO neurons was slightly increased by transfection of hTau40-WT but did not reach significance (*p* = 0.1937). hTau40-ΔPAD (*p* > 0.999) or hTau40-T205A (*p* > 0.999) transfection did not increase the number of primary neurites. **(D)** Impaired axonal outgrowth was significantly improved by transfection of hTau40-WT (*p* = 0.0005). By contrast, transfection of hTau40-ΔPAD did not increase axonal outgrowth (*p* = 0.944). After transfection of hTau40-T205A tau KO actually showed less axonal outgrowth than untransfected neurons (*p* = 0.0242). **(E)** Dendritic outgrowth was also increased by transfection with hTau40-WT (*p* = 0.0005). Transfection of hTau40-ΔPAD (*p* > 0.999) or hTau40-T205A (*p* > 0.999) did not increase dendritic outgrowth. **(F)** The total number of branches of tau KO hippocampal neurons transfected with hTau40-WT (*p* > 0.999), hTau40-ΔPAD (*p* = 0.424) or hTau40-T205A (*p* = 0.986) was not increased. **(G)** The number of axonal branches after transfection with hTau40-WT (*p* > 0.999), hTau40-ΔPAD (*p* = 0.093) or hTau40-T205A (*p* = 0.430) was not increased. **(H)** The total length of tau KO neuron axonal and dendritic branches was not increased by transfection with hTau40-WT (*p* = 0.999), hTau40-ΔPAD (*p* = 0.698) or hTau40-T205A (*p* = 0.698). All data was collected after 96 h in culture. N = 101–115 individual neurons analyzed (see Supplementary Tables S4, 5). ****p < 0.0001, ***p < 0.001, **p < 0.01, *p < 0.05.

Multiple sources indicate that tau is important for neurite outgrowth however its mechanism of action is unknown. Exposure of tau N-terminal PAD in growth cones (Figure 2) during development of hippocampal primary neurons strongly suggests that the PAD is involved in delivery of cargoes to these neuronal subdomains. If this is the case then neurite outgrowth should be dependent on the presence and exposure of the PAD. Moreover, previous research indicated that PAD exposure can be regulated by a single phosphorylation at residue T205 (Morris et al., 2021), and co-localization of PAD exposure and pT205 by immunofluorescence (Figure 2; Supplementary Figure S1) also suggest that phosphorylation at this residue is important. To test the dependence of neurite outgrowth on PAD exposure and phosphorylation of residue T205, tau KO neurons were transfected 16–18 h after plating with 0.5 μg of full length hTau40-WT, hTau40-ΔPAD, or hTau40-T205A. Tau proteins had a C-terminal mCherry tag to visualize transfected cells. Cells were fixed 72 h after transfection, at 96 h in culture, and outgrowth was analyzed by tubulin immunoreactivity (Figure 6A).

Transfection of hTau40-WT significantly increased the axonal outgrowth of tau KO neurons cultured on either PLL (Supplementary Figure S3D) or combined PLL + Laminin (Figure 6D). By contrast, hTau40-ΔPAD or hTau40-T205A did not increase axonal outgrowth compared to untransfected tau KO cells (Figure 6D; Supplementary Figure S3D). Interestingly, tau KO neurons cultured on PLL + Laminin and transfected with hTau40-T205A actually had less axonal outgrowth after 96 h in culture than untransfected neurons (Figure 6D). This may suggest that phosphorylation at T205 is also important for additional downstream pathways as well as exposure of PAD and activation of PP1-GSK3β.

Transfection of hTau40-WT also increased the total length of dendritic outgrowth compared to untransfected tau KO neurons (Figure 6E; Supplementary Figure S3E). For cells grown on PLL alone, hTau40-ΔPAD transfection slightly increased dendritic outgrowth but was still not significantly different from untransfected (Supplementary Figure S2). Transfection of hTau40-T205A did not increase dendritic outgrowth compared to untransfected tau KO neurons on either PLL (Supplementary Figure S3E) or PLL + Laminin (Figure 6E) growth substrates. As well as affecting outgrowth length, hTau40-WT transfection also increased the number of primary neurites for neurons grown on PLL (Supplementary Figure S3C). There was also a slight increase in the number of primary neurites of neurons grown on PLL + Laminin after hTau40-WT transfection but the increase did not reach significance (Figure 6C).

Both the number of and length and branches were significantly less for tau KO neurons compared to WT neurons (Figure 5; Supplementary Figures S3F-H) and transfection of hTau40 did not affect the overall length of dendritic or axonal branches (Figure 6; Supplementary Figures S3G, H). However, transfection of hTau40-WT did increase the overall number of branches slightly on both a PLL (Supplementary Figure S3F) or PLL + Laminin (Figure 6F) substrate and this trend may have continued and become significant at a later timepoint. hTau40-ΔPAD or hTau40-T205A transfection did not have any increase in number or length of branches compared to untransfected tau KO neurons (Figure 6; Supplementary Figures S3 F–H). Even without branches being affected, total neurite outgrowth length was significantly longer after hTau40-WT transfection compared to non-transfected tau KO neurons (Figure 6B; Supplementary Figure S3B). The total neurite outgrowth of hTau40-ΔPAD or hTau40-T205A transfected neurons was not significantly different from untransfected tau KO neurons (Figure 6B; Supplementary Figure S3B).

## 4 Discussion

Previously our lab identified a phosphatase activating domain comprising amino acids 2–18, the PAD, at the N-terminal of tau. Exposure of the PAD leads to activation of a PP1-GSK3β signaling pathway resulting in inhibition of anterograde FAT (LaPointe et al., 2009; Kanaan et al., 2011). Studies have demonstrated that PAD exposure is an early event in multiple tauopathies, co-localizing with tau oligomers and AT8 immunoreactivity (Kanaan et al., 2011; Kanaan et al., 2012; Cox et al., 2016; Kanaan et al., 2016), but the question of whether the PAD also has a physiological role was unknown. The selective inhibition of anterograde transport led us to propose that transient, site-specific exposure of the N-terminal PAD, and activation of the PP1/GSK3β signaling cascade, is a mechanism for delivery of select MBO cargos in axons and would therefore be important for neuronal development. In this paper we have established that there is selective exposure of the PAD during hippocampal neuron development at sites of cargo delivery, comparable to the localization of pT205 tau and active dephospho-Ser9-GSK3β. In addition, we have demonstrated that tau PAD exposure and phosphorylation of T205 have a functional role during neurite outgrowth.

Analysis of primary hippocampal cultures by immunostaining revealed that the PAD is exposed throughout development at specific neuronal subdomains such as growth cones. At both early and later developmental timepoints, TNT1 immunoreactivity was observed at the end of growing neurites and within the central portion of growth cones (Figures 2B,C,E) similar to active GSK3β (Morfini et al., 2002; Morfini et al., 2004). Previous studies from our lab concluded that active GSK3β is enriched in growth cones through use of total and inactive phospho-Ser9-GSK3β antibodies (Morfini et al., 2002; Morfini et al., 2004). Using a recently developed antibody which specifically recognizes active dephospho-Ser9-GSK3β (Grabinski and Kanaan, 2016) we were able to confirm that the previous findings that dp-GSK3β is localized in the central region of growth cones (Figures 2A, D) as well as the cell body (Figure 4).

In early development (2 DIV), TNT1 immunoreactivity was enriched along the length of the growing axon but was observed at much lower levels in dendrites and axonal branches (Figure 3A). By seven DIV, PAD exposure was confined to the growth cone regions of the axon but was also visible in some, but not all, dendrites (Figure 3C). This temporal change in localization is significant as hippocampal neurons in culture follow a stereotypical pattern of outgrowth. After a brief multipolar stage, one neurite begins to elongate rapidly whilst the other processes remain quiescent or retract slightly. On approximately day 5 of culture the minor processes resume elongation and acquire dendritic morphology (Fletcher and Banker, 1989). The absence of TNT1 immunoreactivity in minor processes at 2 DIV but its appearance by 7 DIV suggests that the PAD is specifically exposed in actively growing neurites. Moreover these results suggest that, at least during early stages of development, exposure of the PAD is acting in both developing axons and dendrites. Our previous studies found that GSK3β preferentially phosphorylates KLC2 which can associate with homodimers of any of the kinesin heavy chain isoforms (KIF5A, B, or C) to form the conventional kinesin complex (Morfini et al., 2002; DeBoer et al., 2008). Accordingly, the PP1/GSK3β signaling cascade downstream of PAD exposure would be expected to directly target a subset of anterograde FAT cargoes transported by conventional kinesin isoforms containing KLC2. KIF5 motors have been suggested to have functions in both axons and dendrites (Setou et al., 2002; Kanai et al., 2004; Hirokawa et al., 2009), although other studies have found that KIF5 is excluded from dendrites (Jacobson et al., 2006; Karasmanis et al., 2018) and recombinant KIF5-GFP expressed in primary hippocampal neurons preferentially localized to axons (Nakata and Hirokawa, 2003). Conventional kinesin is thought to be involved in transport of mitochondria which are required through the neuron and are especially essential during active outgrowth (Tanaka et al., 1998; Iworima et al., 2016; Smith and Gallo, 2018). There could also be indirect actions of PP1 or GSK3β activation on other kinesin motors. In addition, GSK3β is known to have other phosphorylation substrates in the growth cone such as stathmin 3 and microtubule associated protein 1B (MAP1B) which are involved in modulation of microtubule dynamics (Trivedi et al., 2005; Devaux et al., 2012).

In more mature hippocampal neurons (10 DIV), PAD exposure became limited to domains along the axon (Figure 3D) and there was no TNT1 immunoreactivity in MAP2-positive dendrites. PAD exposure was most prominent in small regions of the axon where it was crossing over a cell body or dendrite (Figure 3D). This is noteworthy as these subdomains are likely to be sites of nascent synapses. These are fluid structures that form rapidly upon axo-dendritic contact in culture (Ahmari and Smith 2002; Washbourne 2015). Interestingly, the protein components needed to form a stable, mature synapse are transported in two classes of vesicles which both display increased pausing at nascent synapses (Ahmari et al., 2000; Bury and Sabo, 2011). Furthermore, experiments indicated that this pausing is most likely due to both vesicle types responding to a common signal (Bury and Sabo, 2011). Pre-synaptic membrane proteins such as synapsin-I, SNAP25, syntaxin-1B have been shown to be transported by conventional kinesin (Szodorai et al., 2009). These results suggest that exposure of the PAD and activation of PP1-GSK3β may facilitate delivery of presynaptic components at nascent synapses.

Since the N-terminal PAD was only detectable at specific subdomains of developing hippocampal neurons, it follows that there must be strict regulation to control its exposure. Tau conformation is known to be regulated through phosphorylation and we have also shown that tau phosphorylation can regulate downstream signaling pathways (Jeganathan et al., 2008; Bibow et al., 2011; Kanaan et al., 2011; Morris et al., 2021). Building on data from Morris et al., 2021 where we demonstrated that pseudophosphorylation of hTau40-T205E is sufficient to inhibit anterograde FAT through PAD exposure and GSK3β activation, we explored the localization of T205 phosphorylation in primary hippocampal neurons observing similar localization to PAD exposure throughout development (Figure 3; Supplementary Figure S1). GSK3β was more widely distributed, but the active form of GSK3β exhibited enrichment in cell regions similar to TNT1 and pT205 (Figure 4). The localization of pT205 at sites of PAD exposure, combined with previous results, supports the hypothesis that phosphorylation of T205 is linked to exposure of the PAD at specific sites during development. This hypothesis is further supported by the dynamics of T205 phosphorylation. pT205 is rapidly dephosphorylated in mouse brain lysates with over 50% of the signal lost within the first 60 s after death indicating this is a dynamic phosphorylation site (Wang et al., 2015). Moreover, unlike most tau phosphoresidues, pT205 is preferentially dephosphorylated by PP1 (Liu et al., 2005) suggesting there may be negative feedback loop for PAD exposure. This is essential as overexposure of the PAD is linked to multiple tauopathies (Kanaan et al., 2011; Kanaan et al., 2016; Combs and Kanaan, 2017) and overexpression of 6D tau, an N-terminal tau construct that cannot fold, in primary hippocampal neurons results in axon degeneration (Morris 2021).

The location of PAD exposure and T205 phosphorylation during hippocampal neuron development is consistent with our hypothesis that site-specific, regulated exposure of the PAD is a mechanism for delivery of specific MBOs from kinesin. Since axonal transport is essential for neurite outgrowth, we investigated the function of PAD exposure and T205 phosphorylation in tau knockout neurons. As previously shown (Dawson et al., 2001), we found tau knockout neurons to have inhibited outgrowth compared to WT neurons (Figure 5; Supplementary Figure S2). Transfection of full length hTau40-WT into tau KO hippocampal neurons significantly increased their axonal and dendritic outgrowth compared to untransfected cells (Figure 6, Supplementary Figures S3D, E). By contrast transfection of hTau40-ΔPAD or hTau40-T205A did not increase outgrowth compared to untransfected tau KO neurons (Figure 6, Supplementary Figures S3D, E). These results clearly demonstrated that exposure of the N-terminal PAD during hippocampal neuron development is important for normal neurite outgrowth. Furthermore, phosphorylation at T205 appears to be necessary for exposure of the PAD. The deficit and improvement of dendritic outgrowth also supports a role for PAD exposure and T205 phosphorylation in dendritic growth cones as was suggested by TNT1 and pT205 immunostaining.

Interestingly, tau KO hippocampal neurons also had deficits in axonal and dendritic branching compared to WT neurons (Figure 5; Supplementary Figures S2F–H) which were not significantly improved by hTau40 transfection (Figure 6; Supplementary Figures S3 F–H). However, the total number and total length of branches was increased by hTau40-WT transfection although it did not reach significance. This appears to have been driven by a slight increase in the length of dendritic branches since the length of axonal branches was unchanged by transfection (Figure 6G; Supplementary Figure S3G). One reason for the limited increase in branch number and length may be the tau isoform used for transfection. The longest isoform of tau (hTau40; 2N4R) was selected to follow on from previously published experiments (Kanaan et al., 2011; Morris et al., 2021). However, in mice, fetal tau primarily consists of the 0N3R isoform until after birth, at which time expression gradually switches to 4R isoforms (Takuma et al., 2003). Moreover, even after this developmental switch, 2N4R is restricted to the soma and does not become enriched in the axon until adulthood (Liu and Götz, 2013). Further experiments will be required to explore the function of tau in neurite branching, as well as the implications of isoform specific functions.

*In vivo*, tau knockout in mice appears to cause very few overt deficits. Initially the only noted difference was a decreased number and density of microtubules in unmyelinated cerebellar parallel fibers (Harada et al., 1994). However, more rigorous testing showed that tau knockout mice had deficits in motor coordination as well as learning impairments in contextual fear conditioning (Ikegami et al., 2000). By contrast, in humans, tau deficiency is implicated in neurodevelopmental delay. Patients with microdeletion at chromosome 17q21.31, which contains the MAPT gene, all display learning difficulties amongst other symptoms (Shaw-Smith et al., 2006; Dubourg et al., 2011). Acute knockdown of tau in mice also had striking phenotypes of inhibited neurite outgrowth *in vitro* and inhibition of neuronal migration and dendritic development *in vivo* (Caceres and Kosik, 1990; Caceres et al., 1991; Sapir et al., 2012). Some functions of tau appear to be compensated by MAP1B in axons and MAP2 in dendrites (DiTella et al., 1996; Takei et al., 2000; Liu et al., 2019). In particular, tau and MAP1B appear to act synergistically as double knockout mice had exacerbated inhibition in axonal elongation and neuronal migration (DiTella et al., 1996; Takei et al., 2000). Neither MAP1 nor MAP2 contain the PAD, however they could have similar scaffolding functions. For example, PP2A/Bα and Fyn interact with proline rich motifs in both MAP2 and tau (Sontag et al., 2012). Furthermore, perfusion of MAP2c into the squid axoplasm model can inhibit anterograde FAT, identical to tau PAD, through an unknown mechanism (S.T. Brady, unpublished data). A previous study in our lab found that inhibition of Cdk5 can also result in activation of PP1-GSK3β which may represent an independent pathway to regulate cargo delivery in growth cones (Morfini et al., 2004).

Overall the results presented in this paper support our hypothesis that activation of a PP1/GSK3β by regulated site-specific exposure of the N-terminal tau PAD is a mechanism for cargo delivery at select subdomains such as growth cones and nascent synapses and is thereby important during neurite outgrowth. Efficient FAT is essential for development and maintenance of neurons, and the selective delivery of cargoes is likely to involve multiple intersecting mechanisms. It was reported that there is local control of PP1 and GSK3β activity at sites of cargo delivery but mechanisms for activation were unknown (de Waegh et al., 1992; Hsieh et al., 1994; Morfini et al., 2002; Morfini et al., 2004). Exposure of the PAD represents one pathway in developing neurons.

## Data Availability

The raw data supporting the conclusions of this article will be made available by the authors, without undue reservation.

## Supplementary material

Supplementary Table S1 Morris and Brady Supplemental Figures and Tables.DOCX.
