# Supplementary material for: Tau phosphorylation and PAD exposure in regulation of axonal growth
Source: Front Cell Dev Biol. 2023 Jan 18;10:1023418. doi: 10.3389/fcell.2022.1023418 (PMC9893789; doi:10.3389/fcell.2022.1023418)
Supplement: Supplementary file 1 [file Table1.DOCX]

**Supplementary Figures**


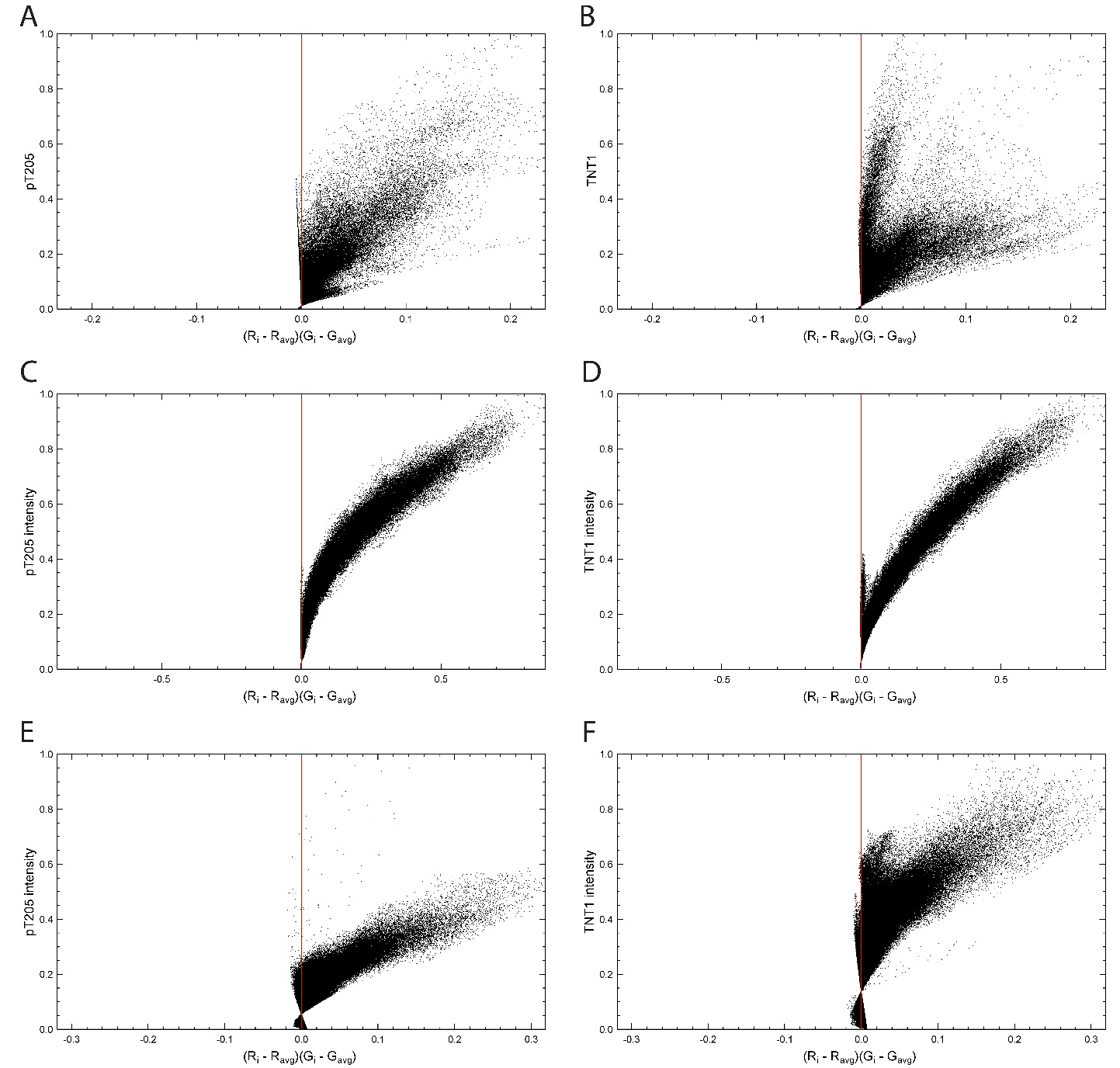


**Figure S1: Colocalization analysis of PAD exposure (TNT1) and pT205 in growth cones and axons.** Pixel overlap of pT205 tau (green) and TNT1 (red) in Fig 2C (2DIV growth cone; **A, B**), Fig 2E (10DIV growth cone; **C, D**), and Fig 3D (10DIV cell body and axon; **E, F**) was analyzed by Intensity Correlation Analysis (ICA) using the JACoP plugin for ImageJ (Bolte and Cordelières, 2006). The ICA method, also known as Li’s approach (Li et al., 2004), is a variation of Pearson’s colocalization correlation with less bias towards high staining intensities. Positive values of (R_i_ – R_avg_)(G_i_ – G_avg_) indicate colocalization whereas negative values indicate segregation of fluorescent signals. R_i_ = red fluorescent intensity at image voxel, i; R_avg_ = average red fluorescent intensity across all image voxels; G­_i_ = green fluorescent intensity at image voxel, i; G_avg_ = average green fluorescent intensity across all image voxels.


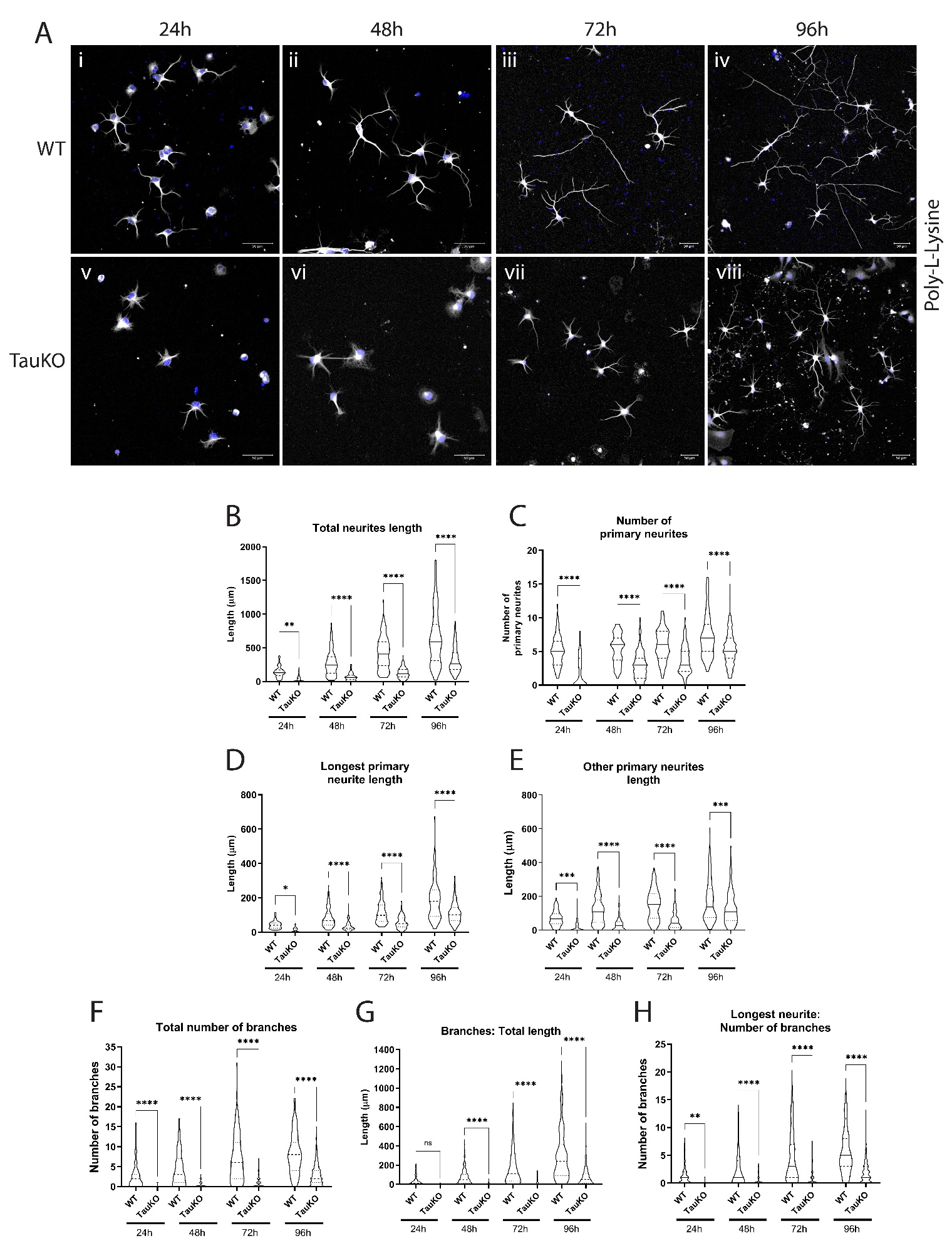


**Figure S2: Tau KO hippocampal neurons cultured on Poly-L-Lysine have impaired neurite outgrowth and branching compared to WT neurons.** Primary hippocampal neurons from WT and tau KO mice were cultured on poly-L-lysine for 24, 48, 72, or 96h followed by staining for β3-tubulin. **(A)** WT hippocampal neurons (i-v) progress through the known stages of neurite outgrowth (Fletcher and Banker, 1989). By contrast, Tau KO hippocampal neurons (v-viii) have less neurite outgrowth and complexity than WT neurons at all timepoints. (Blue: DAPI, White: β3-tubulin) Scale bars: 50 μm. **(B)** Primary hippocampal neurons from tau KO mice have significantly shorter total neurite outgrowth length compared to WT neurons starting from 24h of culture and continuing to 96h of culture. **(C)** The longest primary neurite (axon) was significantly shorter for tau KO hippocampal neurons compared to WT. **(D)** The length of other primary neurites (dendrites) was significantly longer for WT hippocampal neurons compared to tau KO neurons at all timepoints. **(E)** The number of primary neurites remained consistent from 24h – 96h of culture for both WT and tau KO hippocampal neurons and was significantly for WT neurons at all timepoints. **(F)** WT hippocampal neurons have an overall higher number of axonal and dendritic branches than tau KO neurons from 24-96h in culture. **(G)** Primary hippocampal neurons from tau KO mice have significantly shorter secondary and tertiary neurite lengths than WT neurons at the equivalent timepoint in culture. **(H)** WT hippocampal neurons have a significantly higher number of axonal branches than tau KO neurons. N=95-122 individual neurons analyzed (see Supplementary Table 2). ****p<0.0001, ***p<0.001, **p<0.01, *p<0.05

**
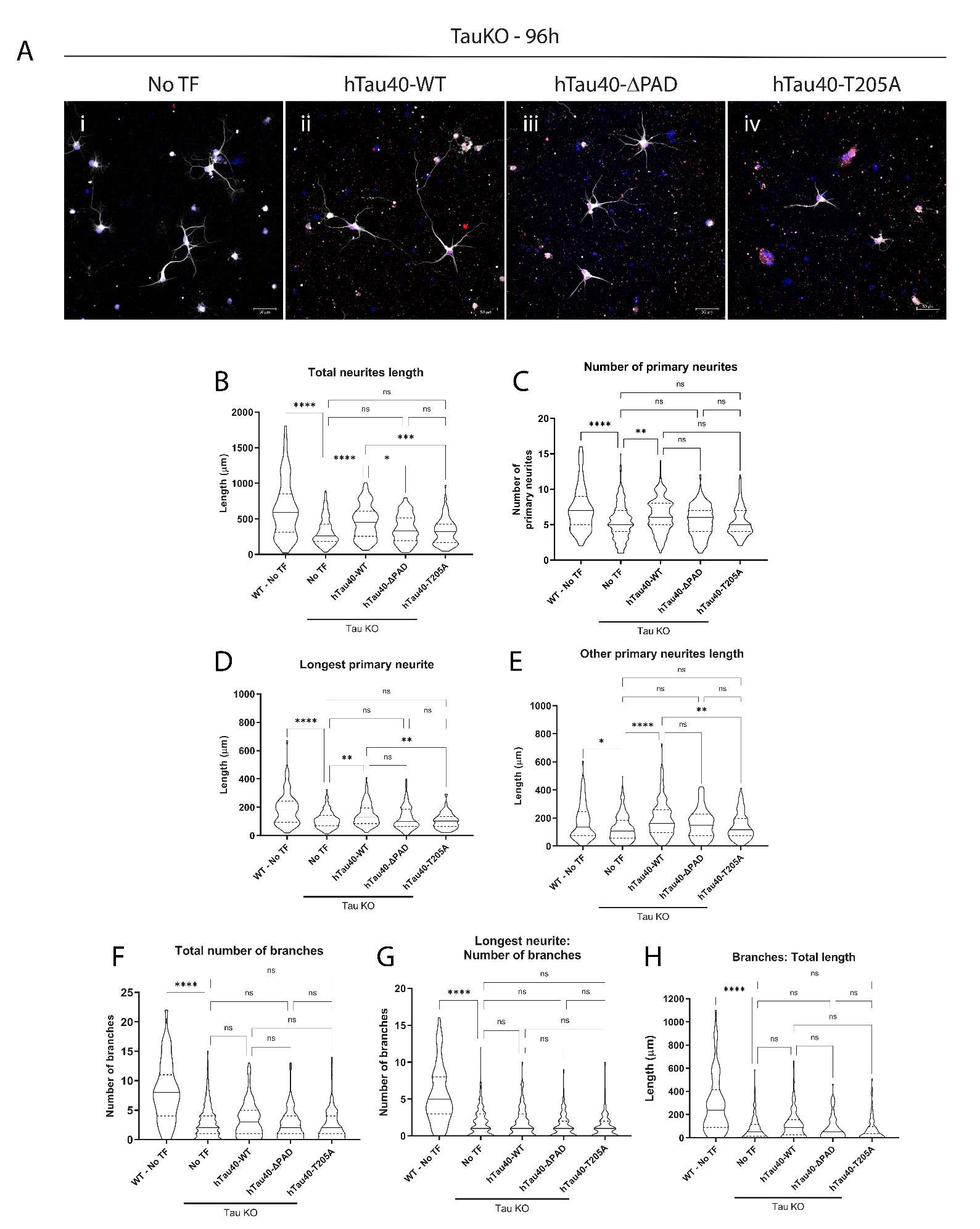
**

**Figure S3:** **Transfection of TauKO hippocampal neurons cultured on poly-L-lysine with hTau40-WT, but not with hTau40-ΔPAD and hTau40-T205A, improves neurite outgrowth of tau KO hippocampal neurons.** Primary hippocampal neurons from tau KO mice cultured on poly-L-lysine were transfected 16-18 h after plating and cultured for a further 72h before fixation and staining for β3-tubulin. **(A)** Tau KO hippocampal neurons had limited neurite outgrowth after 96h (i) but outgrowth was improved after transfection with hTau40-WT (ii). By contrast, transfection of Tau KO hippocampal neurons with hTau40-ΔPAD (iii) or hTau40-T205A (iv) did not improve neurite outgrowth compared to untransfected tau KO hippocampal neurons. (Blue: DAPI, White: β3-tubulin.) Scale bars: 50 μm. **(B)** The total length of all neurites is increased by transfection of hTau40-WT (p<0.0001) but not hTau40-ΔPAD (p=0.967) or hTau40-T205A (p>0.999). **(C)** The number of primary neurites for tau KO neurons was increased by transfection of hTau40-WT (p=0.0089). hTau40-ΔPAD (p>0.999) or hTau40-T205A (p>0.999) transfection did not increase the number of primary neurites. **(D)** Impaired axonal outgrowth was significantly improved by transfection of hTau40-WT (p=0.0013). By contrast, transfection of hTau40-ΔPAD (p=0.986) or hTau40-T205A (p>0.999) did not increase axonal outgrowth. **(E)** Dendritic outgrowth was also increased by transfection with hTau40-WT (p<0.0001). Transfection of hTau40-ΔPAD (p=0.279) or hTau40-T205A (p=0.995) did not increase dendritic outgrowth. **(F)** The total number of branches of tau KO hippocampal neurons transfected with hTau40-WT (p=0.968), hTau40-ΔPAD (p>0.999) or hTau40-T205A (p=0.992) was not increased. **(G)** The number of axonal branches after transfection with hTau40-WT (p>0.999), hTau40-ΔPAD (p=0.977) or hTau40-T205A (p=0.709) was not increased. **(H)** The total length of tau KO neuron axonal and dendritic branches was not increased by transfection with hTau40-WT (p=0.208), hTau40-ΔPAD (p>0.999) or hTau40-T205A (p>0.999). All data was collected after 96h in culture. N=95-151 individual neurons analyzed (see Supplementary table 4). ****p<0.0001, ***p<0.001, **p<0.01, *p<0.05

| **Antigen** | **Host** | **Clone / Conjugate** | **Source** | **Dilution** |
| --- | --- | --- | --- | --- |
| βIII-Tubulin | Rabbit polyclonal | - | Abcam  #ab18207 | 1:1000 |
| βIII-Tubulin | Mouse monoclonal | SDL.3D10 | Sigma  #T8660 | 1:500 |
| MAP2 | Rabbit monoclonal | D5G1 | Cell Signaling Technologies  #8707 | 1:500 |
| Tau | Chicken polyclonal | - | Arigo  #ARG52441 | 1:1000 |
| N-terminal Tau PAD | Mouse monoclonal | TNT1 | Kanaan Lab  (Kanaan et al., 2011; Combs et al., 2016) | 1:10,000 |
| Phospho-T205 Tau | Rabbit monoclonal | EPR2403(2) | Abcam  #ab181206 | 1:1000 |
| Phospho-GSK3β (Ser9) | Rabbit monoclonal | D85E12 | Cell Signaling Technologies  #5558 | 1:100 |
| Non Phospho-GSK3β (Ser9) | Mouse monoclonal | 12B2 | Kanaan Lab  (Grabinski and Kanaan, 2016) | 1:100 |
| Goat anti-mouse | - | AlexaFluor 488 | Invitrogen  #A11029 | 1:800 |
| Goat anti-mouse | - | AlexaFluor 594 | Invitrogen  #A11005 | 1:800 |
| Goat anti-rabbit | - | AlexaFluor 488 | Invitrogen  #A11008 | 1:800 |
| Goat anti-rabbit | - | AlexaFluor 594 | Invitrogen  #A32740 | 1:800 |
| Goat anti-chicken | - | AlexaFluor 647 | Abcam #ab150175 | 1:500 |
| Phalloidin | - | AlexaFluor 647 | Invitrogen #A22287 | 1X |

**Table 1: Monoclonal and polyclonal antibodies used for immunocytochemistry**

| **Laminin** | **WT 24h** | **Tau KO 24h** | **WT 48h** | **Tau KO 48h** | **WT 72h** | **Tau KO 72h** | **WT 96h** | **Tau KO 96h** |
| --- | --- | --- | --- | --- | --- | --- | --- | --- |
| **N** | 101 | 119 | 106 | 103 | 111 | 104 | 96 | 98 |
| **Total neurites length** | 172.7 ± 118.1 | 65.35 ± 69.42 | 347.9 ± 241.9 | 152.3 ± 102.4 | 481.7 ± 298.5 | 229.4 ± 122.7 | 753.5 ± 373.7 | 370.6 ± 197.9 |
| **Longest primary neurite length** | 61.77 ± 43.95 | 28.62 ± 30.66 | 117.4 ± 84.76 | 69.78 ± 51.47 | 146.1 ± 107.9 | 111.0 ± 58.27 | 242.5 ± 173.5 | 131.4 ± 67.23 |
| **Other primary neurites length** | 75.79 ± 59.16 | 32.93 ± 42.81 | 120.2 ± 86.69 | 63.58 ± 59.52 | 171.3 ± 102.4 | 89.31 ± 67.24 | 215.3 ± 135.7 | 145.2 ± 106.1 |
| **Primary neurites: Total number** | 4.67 ± 2.16 | 2.65 ± 2.22 | 5.0 ± 2.14 | 3.42 ± 2.17 | 5.97 ± 2.16 | 4.20 ± 1.98 | 6.93 ± 3.31 | 5.29 ± 2.37 |
| **Branches: Total number** | 2.02 ± 2.30 | 0.32 ± 0.91 | 4.05 ± 4.00 | 0.79 ± 1.30 | 6.05 ± 5.58 | 1.39 ± 1.70 | 7.55 ± 5.23 | 3.01 ± 2.71 |
| **Longest neurite: total branches** | 1.00 ± 1.46 | 0.04 ± 0.20 | 2.23 ± 2.53 | 0.59 ± 1.15 | 3.77 ± 4.51 | 1.02 ± 1.32 | 4.75 ± 3.45 | 1.80 ± 1.91 |
| **Branches: total length** | 34.41 ± 47.56 | 3.25 ± 9.98 | 109.6 ± 133.5 | 12.19 ± 22.46 | 165.2 ± 172.4 | 83.29 ± 96.41 | 295.7 ± 220.8 | 92.65 ± 99.60 |

**Table 2: Summary of morphometric analysis of WT and Tau KO neurons cultured on PLL + Laminin, after 24h, 48h, 72h, and 96h in vitro.** N represents the number of individual neurons counted. Data expressed as mean ± SD.

| **PLL** | **WT 24h** | **Tau KO 24h** | **WT 48h** | **Tau KO 48h** | **WT 72h** | **Tau KO 72h** | **WT 96h** | **Tau KO 96h** |
| --- | --- | --- | --- | --- | --- | --- | --- | --- |
| **N** | 96 | 113 | 110 | 105 | 107 | 99 | 95 | 122 |
| **Total neurites length**  **(μm)** | 142 ± 83.41 | 115 ± 45.29 | 275.2 ± 188.0 | 70.65 ± 58.14 | 422.6 ± 242.5 | 127.0 ± 78.41 | 647.6 ± 407.5 | 321.3. ± 191.4 |
| **Longest primary neurite length**  **(μm)** | 42.81 ± 22.29 | 10.96 ± 12.85 | 82.05 ± 54.92 | 31.68 ± 27.15 | 114.6 ± 63.97 | 56.35 ± 35.54 | 187.9 ± 114.8 | 111.0 ± 58.27 |
| **Other primary neurites length**  **(μm)** | 70.29 ± 44.19 | 20.65 ± 33.73 | 120.2 ± 86.69 | 35.44 ± 41.7 | 149.5 ± 89.25 | 57.4 ± 54.92 | 172.6 ± 126.2 | 127.0 ± 93.19 |
| **Total number: primary neurites** | 5.10 ± 2.18 | 1.90 ± 2.34 | 5.29 ± 2.16 | 2.91 ± 2.04 | 5.96 ± 2.14 | 3.79 ± 2.07 | 7.22 ± 3.25 | 5.37 ± 2.48 |
| **Total number: branches** | 2.39 ± 2.70 | 0.06 ± 0.24 | 4.43 ± 4.13 | 0.34 ± 0.70 | 7.18 ± 5.97 | 0.76 ± 1.25 | 8.20 ± 5.10 | 2.95 ± 2.80 |
| **Longest neurite: total branches** | 1.20 ± 1.60 | 0.04 ± 0.20 | 2.26 ± 2.83 | 0.25 ± 0.64 | 4.63 ± 4.57 | 0.58 ± 1.15 | 5.41 ± 3.85 | 1.70 ± 1.83 |
| **Branches: total length**  **(μm)** | 29.01 ± 38.99 | 0.62 ± 2.61 | 75.05 ± 83.81 | 4.48 ± 10.34 | 156.0 ± 152.4 | 12.19 ± 22.46 | 287.1 ± 248.8 | 83.29 ± 96.41 |

**Table 3: Summary of morphometric analysis of WT and Tau KO neurons cultured on PLL, after 24h, 48h, 72h, and 96h in vitro.** N represents the number of individual neurons counted. Data expressed as mean ± SD.

| **Laminin** | **Tau KO**  **96h** | **hTau40-WT TF**  **96h** | **hTau40-ΔPAD TF**  **96h** | **hTau40-T205A TF**  **96h** |
| --- | --- | --- | --- | --- |
| **N** | 115 | 109 | 101 | 101 |
| **Total neurites length** | 370.6 ± 197.9 | 481.1 ± 216.2 | 329.9 ± 150.8 | 320.8 ± 172.6 |
| **Longest primary neurite length** | 131.4 ± 67.2 | 171.5 ± 103.8 | 116.8 ± 58.6 | 99.9 ± 49.4 |
| **Other primary neurites length** | 145.2 ± 106.1 | 199.2 ± 115.4 | 145.9 ± 91.00 | 144.5 ± 99.1 |
| **Primary neurites: Total number** | 5.29 ± 2.37 | 6.04 ± 2.22 | 5.26 ± 2.12 | 5.26 ± 2.22 |
| **Branches: Total number** | 3.01 ± 2.72 | 3.20 ± 2.80 | 2.17 ± 2.02 | 2.63 ± 2.17 |
| **Longest neurite: total branches** | 1.804 ± 1.913 | 1.688 ± 1.829 | 1.079 ± 1.246 | 1.238 ± 1.471 |
| **Branches: total length** | 92.65 ± 99.60 | 110.4 ± 117.8 | 67.18 ± 73.53 | 76.44 ± 88.66 |

**Table 4: Summary of morphometric analysis of untransfected and transfected Tau KO neurons cultured on PLL + Laminin, after 96h in vitro.** N represents the number of individual neurons counted. Data expressed as mean ± SD.

| **PLL** | **Tau KO**  **96h** | **hTau40-WT TF**  **96h** | **hTau40-ΔPAD TF**  **96h** | **hTau40-T205A TF**  **96h** |
| --- | --- | --- | --- | --- |
| **N** | 124 | 151 | 95 | 99 |
| **Total neurites length** | 321.3 ± 191.4 | 457.2 ± 233.8 | 361.6 ± 196.9 | 321.6 ± 183.7 |
| **Longest primary neurite length** | 111.0 ± 58.27 | 146.7 ± 77.84 | 124.5 ± 82.05 | 104.3 ± 54.31 |
| **Other primary neurites length** | 127.0 ± 93.19 | 192.8 ± 127.5 | 158.7 ± 103.1 | 142.4 ± 94.02 |
| **Primary neurites: Total number** | 5.37 ± 2.48 | 6.28 ± 2.45 | 5.48 ± 2.21 | 5.34 ± 2.11 |
| **Branches: Total number** | 2.95 ± 2.80 | 3.41 ± 2.92 | 2.65 ± 2.61 | 2.49 ± 2.66 |
| **Longest neurite: total branches** | 1.70 ± 1.83 | 1.70 ± 1.88 | 1.35 ± 1.62 | 1.22 ± 1.54 |
| **Branches: total length** | 83.29 ± 96.41 | 117.2 ± 121.6 | 89.61 ± 101.3 | 74.92 ± 98.85 |

**Table 5: Summary of morphometric analysis of untransfected and transfected Tau KO neurons cultured on PLL, after 96h in vitro.** N represents the number of individual neurons counted. Data expressed as mean ± SD.
